# Editorial: Deep facial attribute analysis

**DOI:** 10.3389/fnins.2023.1280831

**Published:** 2023-09-05

**Authors:** Yinghui Kong, Ke Zhang, Li Zhang, Gengshen Wu

**Affiliations:** ^1^Department of Electronics and Communication Engineering, North China Electric Power University, Beijing, China; ^2^Hebei Key Laboratory of Power Internet of Things Technology, North China Electric Power University, Beijing, China; ^3^Department of Computer Science, Royal Holloway, University of London, Egham, United Kingdom; ^4^Faculty of Data Science, City University of Macau, Macao, Macao SAR, China

**Keywords:** facial attribute, age estimation, face recognition, facial expression recognition, facial caricature generation, autistic traits

Face images bear extremely useful social cues, including age, gender, identity, and emotional states, etc. Face attribute is the specific description of the facial features. Face attribute analysis is to analyze the attributes and characteristics of facial images. It has important deployments in many fields. Face attribute analysis based on deep learning techniques has achieved outstanding breakthrough.

As an important physical and social characteristics of human beings, age estimation based on facial images has attracted significant attention, such as cross-age face recognition, harmonious human-computer interaction, image and video retrieval, face-based age prediction, and marketing analysis. Shi et al. summarize modern face-based age estimation methods, and proposed an attention-based convolution (ABC) age estimation framework, namely an improved Swin Transformer with ABC. Their framework improves the accuracy of face age estimation and provides a novel approach for inspiring future investigation.

Face verification and face recognition is to retrieve and identity faces from large-scale face databases or from live surveillance videos based on given samples to provide instant authentication (Hu et al., 2014; Ge et al., 2016). These techniques are widely used because of its effectiveness and efficiency, but the security of face recognition cannot be ignored (Yang et al., 2023).

Facial expression recognition (FER) forms the basis for machines to understand human emotions and detect abnormalities. With the transition of FER from laboratory-controlled to challenging in-the-wild conditions and the recent success of deep learning techniques in various fields, recent deep FER systems generally focus on the important issues: over-fitting caused by a lack of sufficient training data and expression-unrelated variations, such as illumination, head pose and identity bias. Li and Deng (2022) provide a comprehensive survey on deep FER, including datasets and algorithms that provide insights into these intrinsic problems. They review existing novel deep neural networks and related training strategies that are designed for FER based on both static images and dynamic image sequences, and reveal their advantages and limitations. Then extend the survey to include additional related issues and application scenarios, and review the challenges and corresponding opportunities in this field as well as future directions for the design of robust deep FER systems. To meet the requirements of high precision and real-time expression recognition on edge devices, Kong et al. (2022) proposed a real-time facial expression recognition method based on iterative transfer learning and efficient attention network (EAN) for edge resource-constrained scenes. This method can significantly reduce the model complexity and achieve high recognition accuracy rates.

Facial expressions are also used for examination and diagnosis of medical conditions. Meng et al. analyzed that individuals with Autism Spectrum Disorder (ASD) demonstrate a vigilance-avoidance attention pattern toward emotional faces. They employed the eye-tracking technology to examine the characteristics and temporal course of attention bias toward emotional faces in individuals with ATs.

The face attribute editing based on deep learning model is a hot topic of face algorithm and application. In recent years, there are many applications in Internet products, such as face make-up, face age modification, face cartoon avatar generation, face modification and so on. Zhao et al. proposed a style Attention based Global-local Aware GAN for Personalized Facial Caricature Generation. In order to integrate the facial characteristics of a subject, they introduce a landmark-based warp controller for personalized shape exaggeration. To fuse the facial feature with caricature style appropriately, they introduce a style-attention module, which adopts an attention mechanism to reduce the collapsed cases and increase the quality of generated caricatures.

In order to verify the effectiveness of deep learning based facial attribute analysis, various facial databases have been collected and published for algorithm training and testing. But because of the influence of ethnicity, Monteiro et al. provided evidence that facial asymmetry measurements may not be fully comparable across different population, which could potentially affect the reliability of research results carried out with different databases. They verified differences between two face databases, the Chicago Face Database (composed of Asian, Black, Latin, White, and Multiracial subjects) and the LACOP Database (composed of Brazilian subjects). Their work found consistent differences between the two face databases taking into account the ethnicity of the faces. These Results reinforce the need to create and expand multi-ethnic face image databases, allowing for face research in specific populations. Their findings are important not only for face perception research but also for computer vision.

The collection of the studies presented in this Research Topic of Frontiers in Endocrinology will encourage integration and fertilization across disciplines, and promote the further development in this field.

## Author contributions

YK: Funding acquisition, Investigation, Writing—original draft. KZ: Funding acquisition, Writing—review and editing. LZ: Writing—review and editing. GW: Writing—review and editing.

## References

[B1] Ge S. Zhao S. Li C. (2016). Low-resolution face recognition in the wild via selective knowledge distillation. IEEE Trans. Image Process. 28, 2051–2062. 10.1109/TIP.2018.2883743 30507531

[B2] Hu J. Lu J. Tan Y.-P. (2014). “Discriminative deep metric learning for face verification in the wild,” in 2014 IEEE Conference on Computer Vision and Pattern Recognition (CVPR) (Columbus, OH: IEEE). 10.1109/CVPR.2014.242

[B3] Kong Y. Zhang S. Zhang K. Ni Q. Han J. (2022). Real-time facial expression recognition based on iterative transfer learning and efficient attention network. IET Image Process. 16, 1694–1708. 10.1049/ipr2.12441

[B4] Li S. Deng W. (2022). “Deep facial expression recognition: a survey,” in IEEE Transactions on Affective Computing, Vol. 13 (IEEE), 1195–1215. 10.1109/TAFFC.2020.2981446

[B5] Yang X. Liu C. Xu L. Wang Y. Dong Y. Chen N.. (2023). “Towards effective adversarial textured 3d meshes on physical face recognition,” in Proceedings of IEEE Conference on Computer Vision and Pattern Recognition (CVPR) (Vancouver, BC).

